# Expression, Prognosis and Gene Regulation Network of NFAT Transcription Factors in Non-Small Cell Lung Cancer

**DOI:** 10.3389/pore.2021.529240

**Published:** 2021-04-09

**Authors:** Jin Ma, Rao Du, Yan Huang, Wen Zhong, Huan Gui, Chenmei Mao, Xiudao Song, Jun Lu

**Affiliations:** ^1^ Department of Pharmacy, Children’s Hospital of Soochow University, Suzhou, China; ^2^ Clinical Pharmaceutical Laboratory of Traditional Chinese Medicine, Suzhou TCM Hospital Affiliated to Nanjing University of Chinese Medicine, Suzhou, China; ^3^ Department of Haematology, Children’s Hospital of Soochow University, Suzhou, China

**Keywords:** non-small cell lung cancer, NFATs, prognosis, bioinformatics analysis, gene expression

## Abstract

Non-small cell lung cancer (NSCLC) is the leading cause of cancer-related death worldwide. The nuclear factor of activated T cells (NFAT) family is implicated in tumorigenesis and progression in various types of cancer. However, little is known about their expression patterns, distinct prognostic values, and potential regulatory networks in NSCLC. In this study, we comprehensively analyzed the distinct expression and prognostic value of *NFATs* in NSCLC through various large databases, including the Oncomine, UCSC Xena Browser, UALCAN databases, Kaplan–Meier Plotter, cBioPortal, and Enrichr. In lung adenocarcinoma (LUAD) and lung squamous cell carcinoma (LUSC), *NFAT1/2/4/5* mRNA expression levels were significantly decreased and *NFAT3* mRNA expression level was significantly increased. The cBioPortal database analysis showed that the mRNA dysregulation was one of the single most important factors for *NFAT* alteration in LUAD and LUSC and that both LUAD and LUSC cases with the alterations in the mRNA expression of *NFATs* had significantly better overall survival (OS). High expression levels of *NFAT1/2/4/5* were significantly associated with better OS in LUAD, whereas high *NFAT3* expression led to a worse OS. Overexpression of NFAT1/2 predicted better OS in LUSC, whereas high *NFAT5* expression led to a worse OS. The networks for NFATs and the 50 most frequently altered neighbor genes in LUAD and LUSC were also constructed. NFATs and genes significantly associated with *NFAT* mRNA expression in LUAD and LUSC were significantly enriched in the cGMP-dependent protein kinase and Wnt signaling pathways. These results showed that the NFAT family members displayed varying degrees of abnormal expressions, suggesting that NFATs may be therapeutic targets for patients with NSCLC. Aberrant expression of NFATs was found to be associated with OS in the patients with NSCLC; among NFATs, NFAT3/4 may be new biomarkers for the prognosis of LUAD. However, further studies are required to validate our findings.

## Introduction

Since nuclear factor of activated T cells (NFAT) was identified as an activator for the transcription of interleukin-2 in T-cells, there has been increasing evidence linking NFAT proteins to the transcription of a variety of genes [1]. In human, the NFAT family comprises five members, including calcium-regulated isoforms referred as NFAT1 (NFATc2 or NFATp), NFAT2 (NFATc1 or NFATc), NFAT3 (NFATc4), and NFAT4 (NFATc3 or NFATx) and a tonicity-responsive enhancer-binding protein referred as NFAT5 (TonEBP or OREBP) [2]. In the basal state, NFAT proteins are hyperphosphorylated and reside in the cytoplasm; in the stimulated state, they are dephosphorylated and rapidly translocate into the nucleus to promote gene transcription. The calcium-regulated NFAT 1/2/3/4 were activated by increased intracellular calcium levels via dephosphorylation by calcineurin, whereas NFAT5 lacks the calcineurin docking site and is activated by osmotic stress [3]. NFAT proteins were originally found to regulate genes related to the development, activation, and differentiation of immune cells [4], thereby playing critical roles in immune systems. Subsequent studies have indicated that NFAT family members are also expressed in non-immune cells and tissues, thus participating in many normal bodily processes as well as in the development of several diseases including cancer [5]. Since the first study regarding the correlation between NFAT1/5 and cancer cells was published [6], one or more members of the NFAT family have been reported to be dysregulated in numerous cancer types including hepatocellular carcinoma (HCC) [7, 8], breast cancer [9, 10], colorectal cancer [11, 12], and lung cancer (LC) [13–15].

LC remains the leading cause of cancer-related death worldwide [16], with non-small cell lung cancer (NSCLC) accounting for approximately 85% of LC cases [17]. Lung adenocarcinoma (LUAD) and lung squamous cell carcinoma (LUSC) are the two main subtypes of NSCLC, accounting for roughly 40 and 30% of cases, respectively [18]. Despite considerable advancements in diagnostic and treatment methods, the overall 5-year survival rate for LC patients after treatment is lower than that for colon, breast, and prostate cancers at about 15% [19]. The fact that targeted therapy has been successful in a subset of tumors requires a better understanding of the pathological mechanisms by which these oncogenic alterations cause tumorigenesis of NSCLC. Hence, identifying novel prognostic markers and potential drug targets has become imperative for enhancing prognosis and improving targeted therapy for this disease.

Accumulating evidence has indicated the role of NFAT family members in many aspects of cancer including cell proliferation, metastasis, drug resistance, and the tumor microenvironment [3, 5, 20, 21]. NFAT2/3/4/5 act as tumor suppressors in several types of cancers. NFAT2 and NFAT3 induce apoptosis in HCC [6] and glioma cells [22], respectively; NFAT4 deficiency results in the development of mammary gland adenocarcinoma [23]; NFAT5 inhibits invasion and promotes the apoptosis of HCC cells [8]. By contrast, other studies have shown that NFAT3/4/5 can act as oncogenes. NFAT3 and NFAT4 promote the progression of colon cancer [12], and NFAT5 induces the proliferation and migration of renal carcinoma cells [24]. These findings indicate that the precise functions of different NFAT family members in cancer are context-dependent.

To the best of our knowledge, studies on the role of NFAT proteins in LC are lacking. To date, among NFAT family members, NFAT1, NFAT2, and NFAT5 were reportedly associated with LC [14, 15, 25, 26]. However, bioinformatics analysis has not been applied to explore the role of NFAT family members in LC. Using the published analyses of gene expression and variation in copy numbers in thousands of genes, we analyzed the expression and mutations of different NFAT family members in patients with NSCLC to determine their expression patterns, potential regulatory networks, and distinct prognostic values in this disease.

## Results

### Transcriptional Levels of Nuclear Factor of Activated T Cells Family Members in Patients With Lung Cancer

**FIGURE 1:**
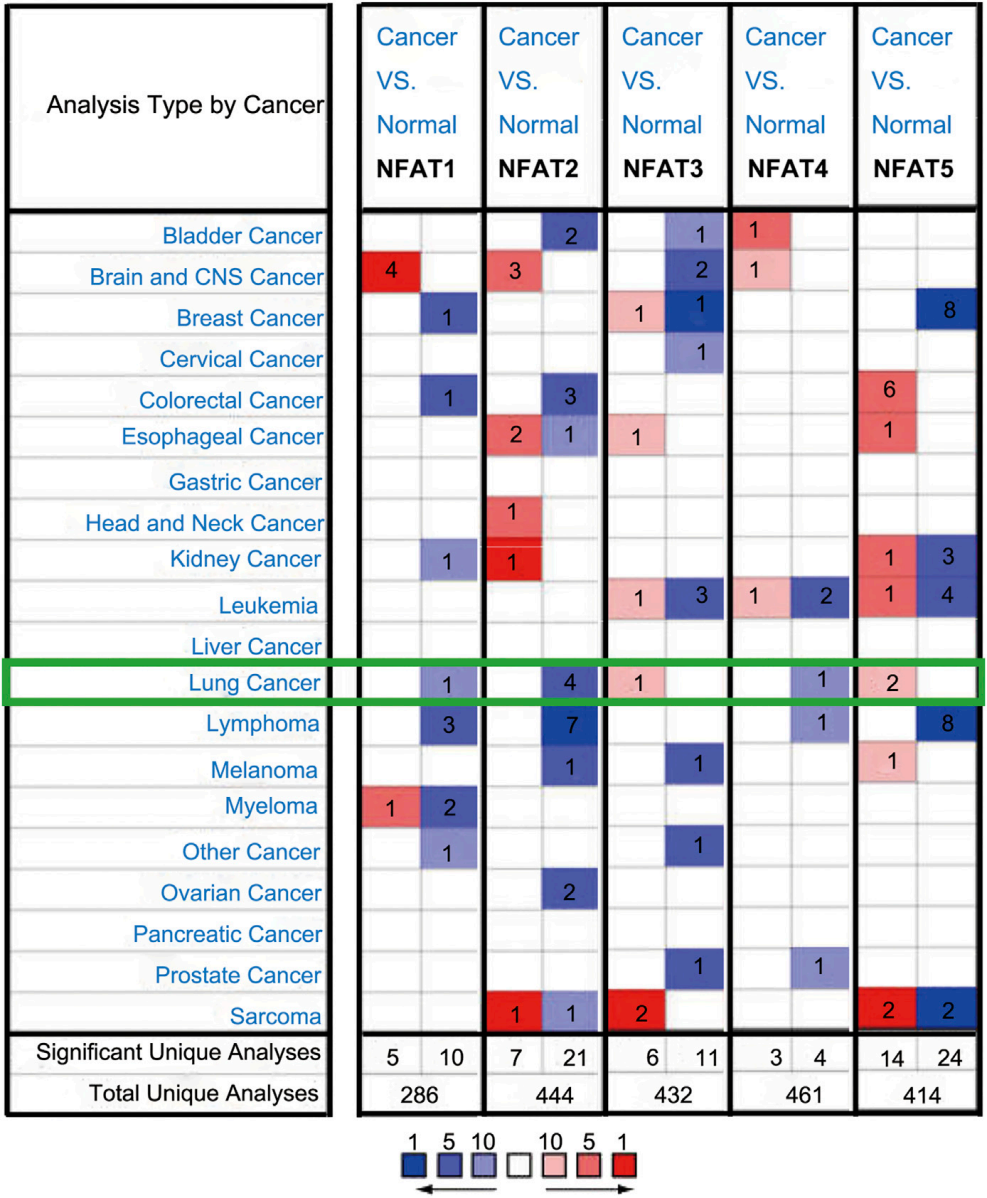
The mRNA expression levels of *NFAT* family members in different cancer types analyzed using the Oncomine database The mRNA expression levels of the five *NFAT* family members (*NFAT1, NFAT2, NFAT3, NFAT4,* and *NFAT5*) between cancer and normal tissues were analyzed using the Oncomine database. The number in the colored cell represents the number of analyses meeting thresholds with statistically significant (*p* < 0.01) mRNA expression. The overexpression (red) or downexpression (blue) of NFATs (different types of cancer vs. corresponding normal tissue) was displayed. Cell color was determined by the best gene rank percentile for the analyses within the cell, and the gene rank was analyzed by percentile of the target gene in the top of all genes measured in each research.

We compared the mRNA expression levels of *NFAT* family members in cancer with those in normal samples using the Oncomine database. As shown in Figure 1, the mRNA expression levels of *NFAT1*, *NFAT2*, and *NFAT4* were downregulated in patients with LC, and those of *NFAT3* and *NFAT5* were upregulated in patients with LC. *NFAT2* mRNA expression was significantly lower in patients with LC in four datasets than that in corresponding normal tissues. In Hou’s dataset [27], the downregulation of *NFAT2* was found in two types of LC compared with normal samples: LUAD with a fold change of −2.023 and LUSC with a fold change of −2.185 (Table 1). The results from Bhattacharjee’s dataset [28] showed that the mRNA expression levels of *NFAT2* in LUSC were also significantly lower than those in normal tissues with a fold change of −4.810. Similarly, in Beer’s dataset [29], *NFAT2* was also significantly downregulated in LUAD with a fold change of −3.714 (Table 1). In addition, the downregulation of *NFAT1* was also found in LUAD patients with a fold change of −2.254 (Table 1). However, In Garber’s dataset [30], the mRNA expression level of *NFAT3* was significantly higher in LUAD with a fold change of 2.538 compared to normal lung tissues (Table 1). Bhattacharjee [28] showed that NFAT4 was downregulated with a fold change of −8.598 in patients with lung carcinoid tumor compared with that in patients with normal lung tissues (Table 1). In Bhattacharjee’s dataset [28], the mRNA expression levels of *NFAT5* in small cell lung carcinoma and lung carcinoid tumor were significantly higher than those in the corresponding normal lung tissues, with their fold changes of 2.175 and 2.421, respectively (Table 1).

**TABLE 1:** Significant changes in NFAT mRNA expression levels between different types of lung cancer and normal lung tissues (Oncomine database).

Gene ID	Types of LC vs. normal	Fold change	*p*-value	*t*-Test	References
NFAT1	Lung squamous cell carcinoma vs. normal	−2.254	1.95E-10	−2.254	[27]
NFAT2	Lung squamous cell carcinoma vs. normal	−4.810	0.003	−3.037	[28]
	Lung adenocarcinoma vs. normal	−3.714	4.38E-6	−5.096	[29]
	Lung adenocarcinoma vs. normal	−2.023	4.19E-13	−8.369	[27]
	Lung squamous cell carcinoma vs. normal	−2.185	1.06E-13	−9.498	[27]
NFAT3	Lung adenocarcinoma vs. normal	2.538	0.002	3.978	[30]
NFAT4	Lung carcinoid tumor vs. normal	−8.598	1.40E-6	−5.577	[28]
NFAT5	Small cell lung carcinoma vs. normal	2.175	0.009	2.572	[28]
	Lung carcinoid tumor vs. normal	2.421	0.002	3.297	[28]

LC, Lung cancer; NFAT, Nuclear Factor of Activated T Cells.

**FIGURE 2:**
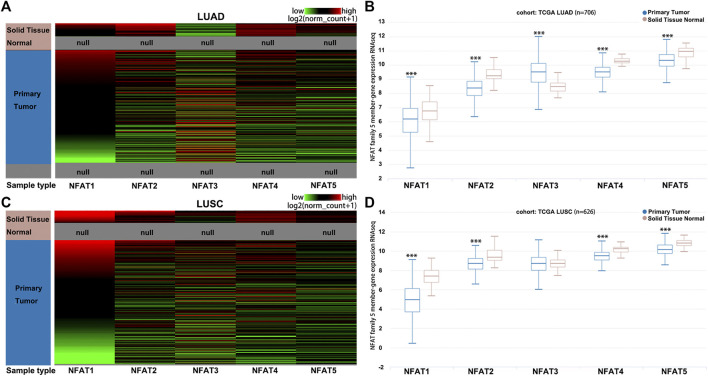
The mRNA expression levels of *NFAT* family members in LUAD and LUSC analyzed using the UCSC Xena browser Heat map **(A, C)** and the corresponding box plots **(B, D)** of *NFAT1*, *NFAT2*, *NFAT3*, *NFAT4* and *NFAT5* between primary tumor tissues in LUAD or LUSC patients and the corresponding normal lung tissues were constructed using the UCSC Xena browser. ***p < 0.001. TCGA, The Cancer Genome Atlas. Lung adenocarcinoma, LUAD; lung squamous cell carcinoma, LUSC.

The hierarchical clustering analysis by using the University of California, Santa Cruz (UCSC) Xena Browser showed that the mRNA levels of *NFAT* family members were basically differentiate in the LUAD (Figure 2A) and LUSC (Figure 2C) samples from the corresponding normal samples. The box plots showed that the mRNA expression levels of *NFAT1*, *NFAT2*, *NFAT4*, and *NFAT5* in LUAD (Figure 2B) and LUSC (Figure 2D) tissues were significantly lower than those in the corresponding normal tissues. The *NFAT3* mRNA expression level in LUAD tissue was significantly higher than that in the corresponding normal tissues (Figure 2B), but no statistical differences had been found in *NFAT3* mRNA expression between LUSC tissue and the corresponding normal tissue (Figure 2D).

### The mRNA Expression Levels of Nuclear Factor of Activated T Cells Family Members in Non-small Cell Lung Cancer Based on Patients’ Pathological Features

**FIGURE 3:**
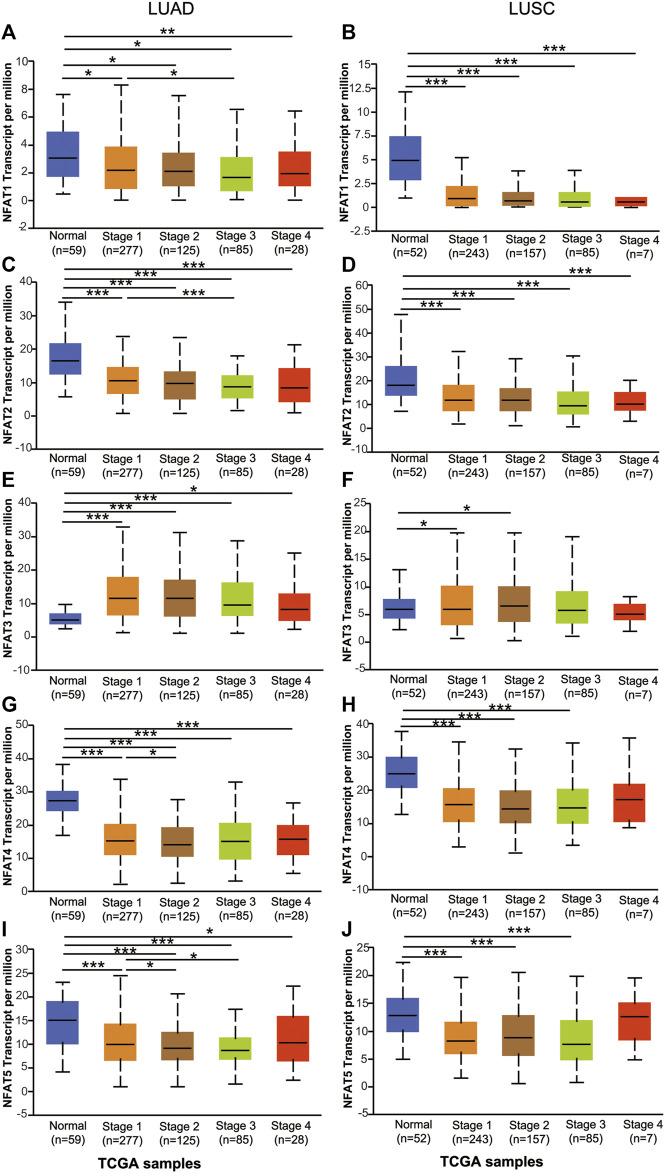
The mRNA expression levels of *NFAT* family members in LUAD and LUSC patients based on individual cancer stage analyzed using the UALCAN database The mRNA expression levels of *NFAT1* **(A, B)**, *NFAT2* **(C, D)**, *NFAT3* **(E, F)**, *NFAT4* **(G, H)** and *NFAT5* **(I, J)** between primary tumor tissues in LUAD or LUSC patients with stage 1, 2, 3 or 4 tumors and the corresponding normal lung tissues were analyzed using the UALCAN database. *p < 0.05, ***p < 0.001. TCGA, The Cancer Genome Atlas. Lung adenocarcinoma, LUAD; lung squamous cell carcinoma, LUSC.

Using the UALCAN database, we further compared the mRNA expression levels of *NFAT* family members between primary tumor tissues in NSCLC patients including LUAD and LUSC and the corresponding normal tissues based on individual pathological stages [1–4] and smoking habits. As shown in Figure 3, the expression levels of *NFAT1*, *NFAT2*, *NFAT4*, and *NFAT5* in all stages of LUAD were significantly lower than those in the corresponding normal tissues, particularly for stage 2 and 3 (Figures 3A,C,G,I). The expression levels of *NFAT1*, *NFAT2*, and *NFAT5* in LUAD stage 3 were significantly lower than that of LUAD stage 1, and the expression levels of *NFAT4* and *NFAT5* in LUAD stage 2 were significantly lower than that of LUAD stage 1 (Figures 3A,C,G,I). The *NFAT3* expression in all stages in LUAD was significantly higher than that in normal tissue (Figure 3E). In LUSC, the expression levels of *NFAT1* and *NFAT2* in all stages were significantly lower than those in the corresponding normal tissues, and the expression levels of *NFAT4* and *NFAT5* in LUSC stage 1, 2 and 3 were significantly lower than that those in the corresponding normal tissues (Figures 3B,D,H,J). Interestingly, LUSC stages 1 and 2 had significantly higher expression in *NFAT3* in comparison with normal tissue (Figure 3F). The expression levels of individual NFAT family members in LUSC did not significantly differ based on tumor stages.

**FIGURE 4:**
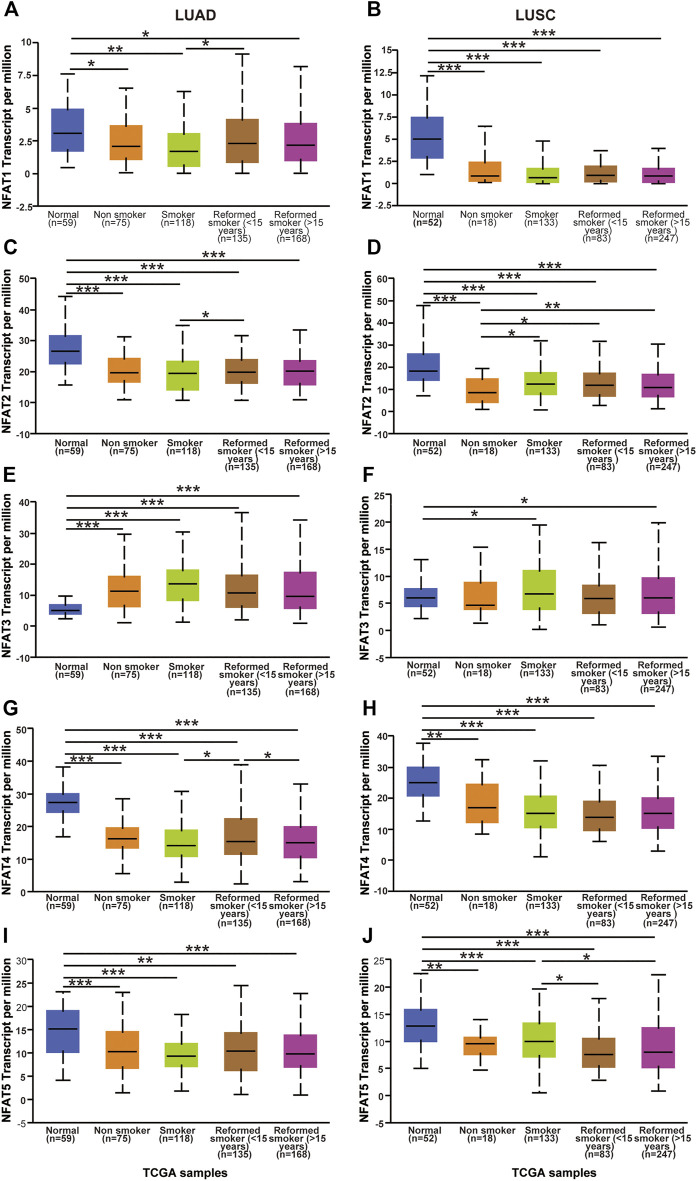
The mRNA expression levels of *NFAT* family members in LUAD and LUSC patients based on patient’s smoking habits analyzed using the UALCAN database The mRNA expression levels of *NFAT1* **(A, B)**, *NFAT2* **(C, D)**, *NFAT3* **(E, F)**, *NFAT4* **(G, H)** and *NFAT5* **(I, J)** in normal individuals or in LUAD and LUSC patients based on patient’s smoking habits were analyzed using the UALCAN database. *p < 0.05, **p < 0.01, ***p < 0.001. TCGA, The Cancer Genome Atlas. Lung adenocarcinoma, LUAD; lung squamous cell carcinoma, LUSC.

We next assessed the expression levels of the NFAT family in LUAD and LUSC based on smoking habits. Regarding LUAD patients based on smoking habits, the expression levels of *NFAT2*, *NFAT4*, and *NFAT5* in all categories were significantly lower than those in the corresponding normal tissues, whereas the *NFAT3* expression level in all categories was significantly higher than that in normal tissue (Figures 4C,E,G,I). The *NFAT1* expression in all categories showed a decrease compared to normal, which was significant for smokers, non-smokers, and reformed smokers (>15 years) (Figure 4A). In LUAD, the expression levels of *NFAT1* and *NFAT4* were significantly higher in reformed smokers (<15 years) compared to smokers (Figures 4A,G). The *NFAT4* expression level was significantly lower in reformed smokers (>15 years) compared to reformed smokers (<15 years) (Figure 4G). Regarding LUSC patients based on smoking habits, the expression levels of *NFAT1*, *NFAT2*, *NFAT4*, and *NFAT5* in all categories were significantly lower than those in the corresponding normal tissues (Figures 4B,D,H,J). The *NFAT3* expression in smokers and reformed smokers (>15 years) showed a significant increase compared to normal (Figure 4F). The *NFAT2* expression level in smokers [smokers, reformed smokers (<15 years), and reformed smokers (>15 years)] was significantly higher compared to non-smokers (Figure 4D). The *NFAT5* expression level was significantly higher in reformed smokers (<15 years) and reformed smokers (>15 years) compared to smokers (Figure 4J).

### Alteration Frequency of Nuclear Factor of Activated T Cells Family Members in Non-small Cell Lung Cancer

**FIGURE 5:**
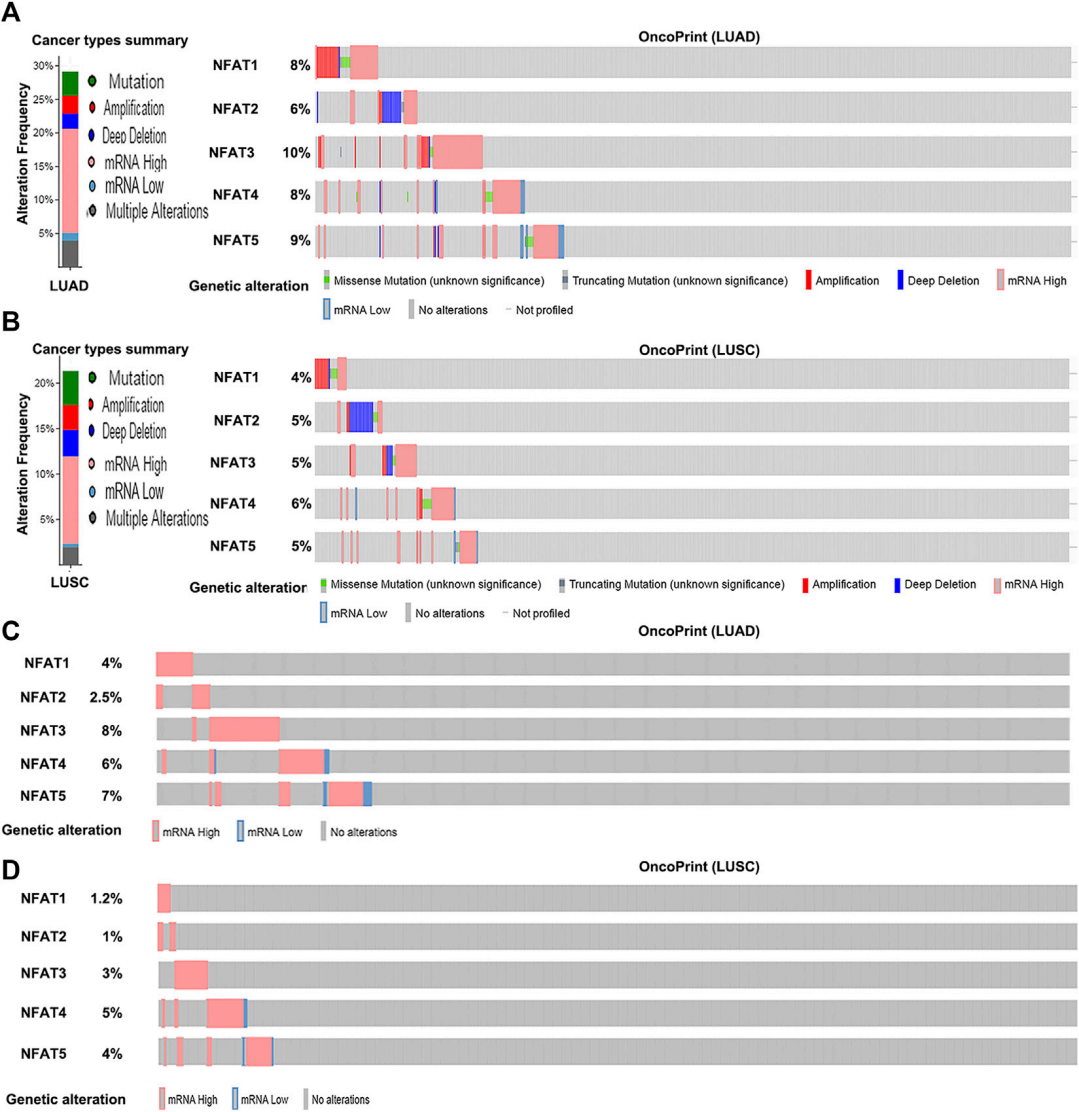
Alteration frequency of NFAT family members in LUAD and LUSC analyzed using the cBioPortal database Cancer types summary and oncoPrint in the cBioPortal represent the proportion and distribution of cases with NFAT family members in LUAD **(A)** and LUSC **(B)**. Alterations in *NFAT* mRNA expression (RNA Seq V2 RSEM) in LUAD **(C)** and LUSC **(D)** were analyzed. Lung adenocarcinoma, LUAD; lung squamous cell carcinoma, LUSC.

The genetic alteration frequencies of NFAT family members in LUAD and LUSC were evaluated using the cBio Cancer Genomics Portal (cBioPortal). A total of 29.18% of LUAD (586 patients; Figure 5A) and 25.28% of LUSC clinical cases (178 patients; Figure 5B) were found to have alterations in NFAT family members. Given the high frequency of NFAT gene alterations, their expression may be dysregulated. Indeed, mRNA dysregulation was more prominent in LUAD (16.72% clinical cases) and LUSC (7.86% clinical cases). Furthermore, NFAT mRNA levels were upregulated in 91 LUAD cases (15.53%) and 13 LUSC cases (7.3%). We further analyzed the alternations in mRNA expression of NFAT isoforms in LUAD and LUSC using RNA Seq V2 RSEM. The results showed that the percentages of mRNA alterations in *NFAT1*, *NFAT2*, *NFAT3*, *NFAT4*, and *NFAT5* genes in patients with LUAD (515 patients) were 4, 2.5, 8, 6, and 7%, respectively (Figure 5C); and in those with LUSC (501 patients) were 1.2, 1, 3, 5, and 4, respectively (Figure 5D).

### Relationship Between the Alterations in the mRNA Expression of *Nuclear Factor of Activated T Cells* and Prognosis in Non-small Cell Lung Cancer

**FIGURE 6:**
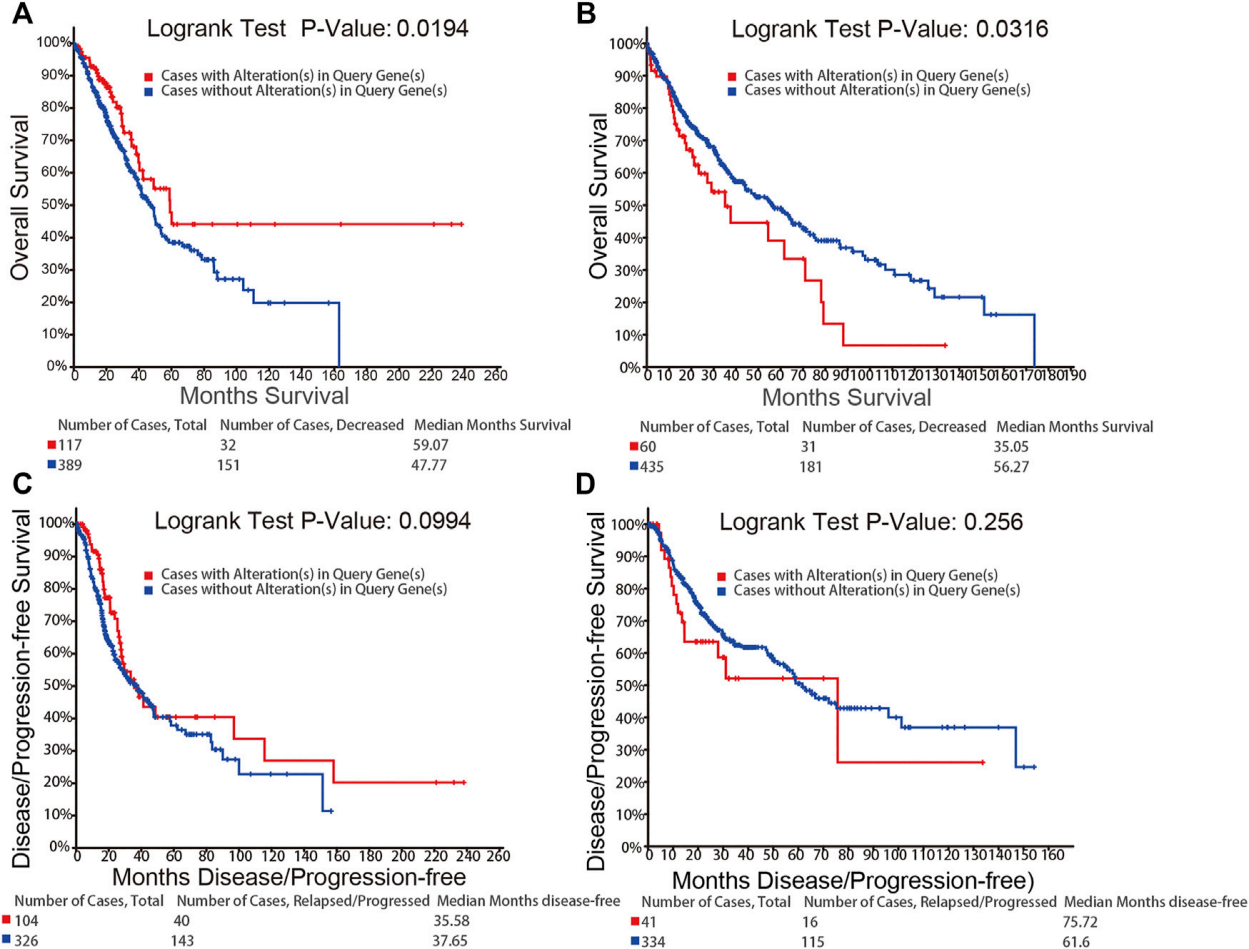
Relationship between the alterations in the mRNA expression of *NFATs* and prognosis in LUAD and LUSC analyzed using the cBioPortal database OS of LUAD **(A)** and LUSC **(B)** patients with *NFAT* mRNA alterations (red) and without *NFAT* mRNA alterations (blue) were analyzed. DFS/PFS of LUAD **(C)** and LUSC **(D)** patients with *NFAT* mRNA alterations (red) and without *NFAT* mRNA alterations (blue) were analyzed. Lung adenocarcinoma, LUAD; lung squamous cell carcinoma, LUSC.

We used the cBioPortal to further study the relationship between the alterations in the mRNA expression of *NFATs* and prognosis. The alterations in the mRNA expression of NFAT isoforms based on the mRNA expression z-scores (RNA Seq V2 RSEM) include the two types, mRNA High (mRNA expression z-Scores > mean + 2.0) and mRNA Low (mRNA expression z-Scores < mean − 2.0). Kaplan–Meier plots were used to compare overall survival (OS) and disease/progression-free survival (DFS/PFS) in LUAD or LUSC cases with and without the alterations in the mRNA expression of NFATs. As shown in Figure 6A, LUAD cases with the alterations of *NFAT* mRNA expression had significantly better OS than those without; however, the DFS/PFS was not significantly different (Figure 6C). LUSC cases with the alterations of *NFAT* mRNA expression exhibited significantly worse OS compared with the cases without the alterations of *NFAT* mRNA expression (Figure 6B), and the DFS/PFS was not significantly different here as well (Figure 6D).

### Correlation Between Overall Survival and the mRNA Levels of Individual Nuclear Factor of Activated T Cells Family Members in Non-small Cell Lung Cancer Patients

**FIGURE 7:**
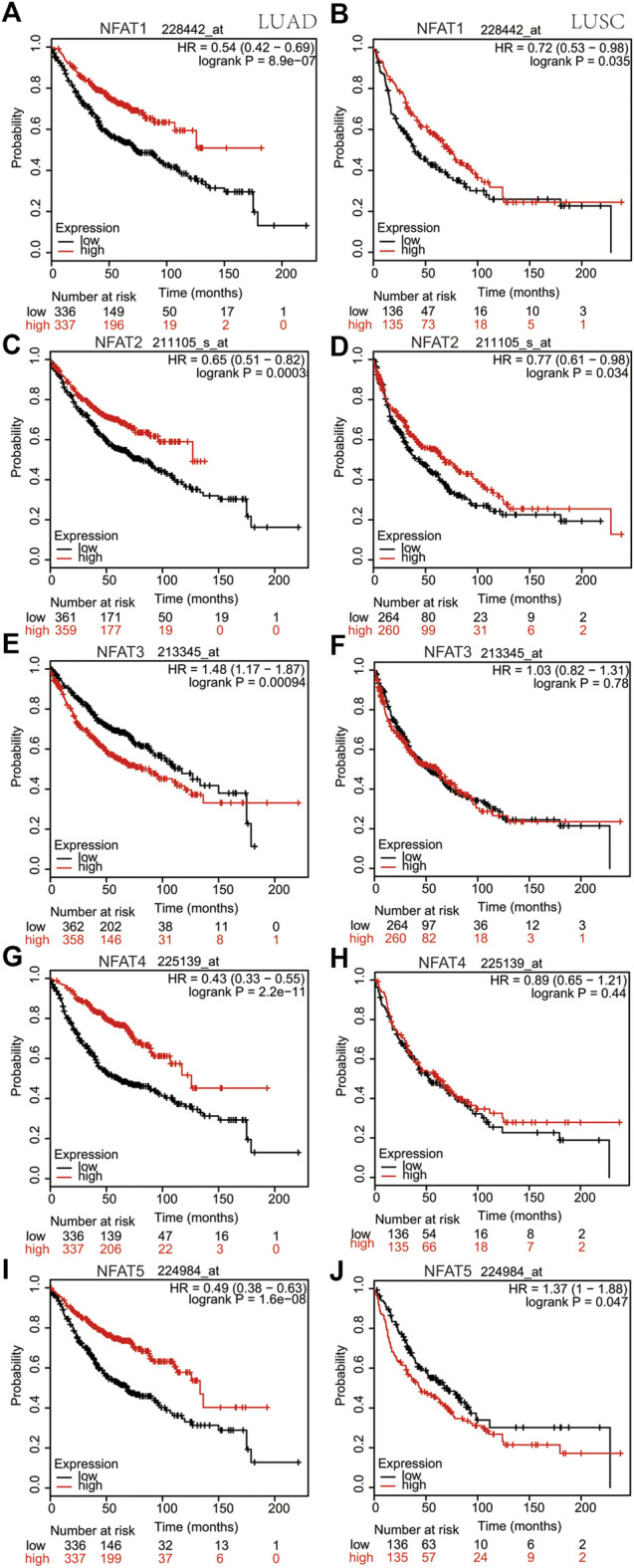
Correlation between overall survivals and the mRNA levels of individual NFAT family members in LUAD and LUSC patients analyzed using the Kaplan-Meier plotter database The OS curve comparing patients with high (red) and low (black) *NFAT* mRNA expression in LUAD and LUSC was plotted from the Kaplan-Meier plotter database. HR, Hazard Ratio. Lung adenocarcinoma, LUAD; lung squamous cell carcinoma, LUSC.

Using the cBioPortal database, survival analysis of alterations in NFAT mRNA expression in LUAD and LUSC patients demonstrated that these alterations were significantly associated with OS but not with DFS/PFS. The Kaplan-Meier plotter online analysis tool was used to further study the OS for LUAD and LUSC patients according to the low or high mRNA expression of *NFAT* family members in The Cancer Genome Atlas (TCGA) database. The high mRNA expression levels of *NFAT1/2/4/5* were significantly associated with a better OS in LUAD patients (Figures 7A,C,G,I), whereas the high *NFAT3* mRNA expression was significantly associated with a worse OS in LUAD patients (Figure 7E). In LUSC patients, the high mRNA expression levels of *NFAT1/2* were significantly associated with a better OS (Figures 7B,D), whereas the high *NFAT5* mRNA expression was significantly associated with a worse OS (Figure 7J). The mRNA expression levels of NFAT3/4 in LUSC patients were not significantly associated with OS (Figures 7F,H).

### Prognostic Values of Nuclear Factor of Activated T Cells Family Members in Non-small Cell Lung Cancer Patients With Different Clinicopathological Features

We further analyzed the effect of high vs. low NFAT expression on the OS for the LUAD and LUSC patients with different clinicopathological characteristics including clinical stage and smoking history. In Table 2, we found that the high mRNA expression levels of *NFAT1/2/4/5* were significantly associated with a better OS in LUAD patients with stage 1. The high mRNA expression of *NFAT4* was significantly correlated with a better OS in stage 2 LUAD patients. In LUSC, overexpression of *NFAT1* was significantly associated with a better OS in stage 1 patients. The increased expression levels of *NFAT2*, *NFAT4*, and *NFAT5* were significantly correlated with a better OS in stage 2 LUSC patients. Table 3 showed prognostic significance between NFAT mRNA expression and smoking history in NSCLC patients including LUAD and LUSC. The high mRNA expression of *NFAT1* was significantly associated with a better OS in LUAD patients with smoking habit. The high mRNA expression levels of *NFAT4/5* were significantly associated with a better OS in both smoking and no smoking LUAD patients. No significant association with OS was identified for any of the NFAT members in LUSC patients with smoking history.

**TABLE 2:** The correlation of NFAT family members with tumor stages of NSCLC patients (Kaplan-Meier plotter database).

Type	Gene ID	Stages	Low (cases)	High (cases)	HR (95%CI)	Logrank *p*
LUAD	NFAT1	1	173	173	0.29 (0.18–0.47)	3.6e−08***
		2	59	59	0.77 (0.45–1.31)	0.34
		3	10	11	2.81 (0.75–10.49)	0.11
	NFAT2	1	185	185	0.36 (0.23–0.55)	1.4e−06***
		2	68	68	0.87 (0.53–1.42)	0.58
		3	12	12	0.81 (0.3–2.19)	0.68
	NFAT3	1	186	184	1.32 (0.9–1.95)	0.15
		2	68	68	1.35 (0.82–2.22)	0.24
		3	12	12	1.1 (0.37–3.24)	0.86
	NFAT4	1	173	173	0.3 (0.19–0.47)	5.5e−08***
		2	59	59	0.42 (0.24–0.74)	0.0017**
		3	10	11	2.01 (0.6–6.75)	0.25
	NFAT5	1	173	173	0.28 (0.18–0.45)	9e−09***
		2	59	59	0.6 (0.35–1.03)	0.063
		3	10	11	1.33 (0.39–4.59)	0.65
LUSC	NFAT1	1	38	36	0.55 (0.3–1)	0.046*
		2	20	19	0.67 (0.25–1.8)	0.42
		3	10	10	1.48 (0.56–3.91)	0.43
	NFAT2	1	86	86	0.67 (0.43–1.02)	0.062
		2	50	50	0.49 (0.26–0.91)	0.021*
		3	22	21	0.99 (0.5–1.95)	0.97
	NFAT3	1	87	85	0.98 (0.64–1.51)	0.94
		2	51	49	0.75 (0.41–1.38)	0.36
		3	22	21	0.9 (0.46–1.78)	0.77
	NFAT4	1	37	37	0.63 (0.35–1.15)	0.13
		2	20	19	0.34 (0.11–0.98)	0.037*
		3	10	10	1.04 (0.38–2.83)	0.94
	NFAT5	1	37	37	1.36 (0.74–2.5)	0.32
		2	20	19	5.28 (1.5–18.55)	0.0037**
		3	10	10	1.79 (0.67–4.83)	0.24

Low/high (cases): low/high expression of the corresponding gene (patient number). LUAD, lung adenocarcinoma; LUSC, lung squamous cell carcinoma; NFAT, nuclear factor of activated T cells. *p < 0.05, **p < 0.01, ***p < 0.001.

**TABLE 3:** The correlation of NFAT family members with smoking history of NSCLC patients (Kaplan-Meier plotter database).

Type	Gene ID	Smoking history	Low (cases)	High (cases)	HR (95%CI)	Logrank *p*
LUAD	NFAT1	Never smoked	70	70	0.87 (0.39–1.98)	0.75
		Smoked	116	115	0.59 (0.36–0.97)	0.036*
	NFAT2	Never smoked	72	71	0.53 (0.23–1.22)	0.13
		Smoked	123	123	0.68 (0.42–1.09)	0.11
	NFAT3	Never smoked	72	71	1.49 (0.66–3.37)	0.33
		Smoked	124	122	1.17 (0.74–1.87)	0.5
	NFAT4	Never smoked	70	70	0.25 (0.09–0.66)	0.0026**
		Smoked	116	115	0.43 (0.26–0.72)	0.00088***
	NFAT5	Never smoked	70	70	0.19 (0.06–0.55)	0.00064***
		Smoked	116	115	0.4 (0.24–0.67)	0.00032***
LUSC	NFAT1	Smoked	29	29	0.52 (0.21–1.27)	0.15
	NFAT2	Smoked	122	122	0.72 (0.5–1.04)	0.08
	NFAT3	Smoked	122	122	1.05 (0.73–1.52)	0.79
	NFAT4	Smoked	29	29	0.46 (0.19–1.13)	0.082
	NFAT5	Smoked	29	29	0.52 (0.21–1.29)	0.15

Low/High (cases): low/high expression of the corresponding gene (patient number). LUAD, lung adenocarcinoma; LUSC, lung squamous cell carcinoma; NFAT, nuclear factor of activated T cells. *p < 0.05, **p < 0.01, ***p < 0.001.

### Correlations Between Nuclear Factor of Activated T Cells Family Members and Network of Nuclear Factor of Activated T Cells and Their 50 Most Frequently Altered Neighboring Genes in Non-small Cell Lung Cancer

**FIGURE 8:**
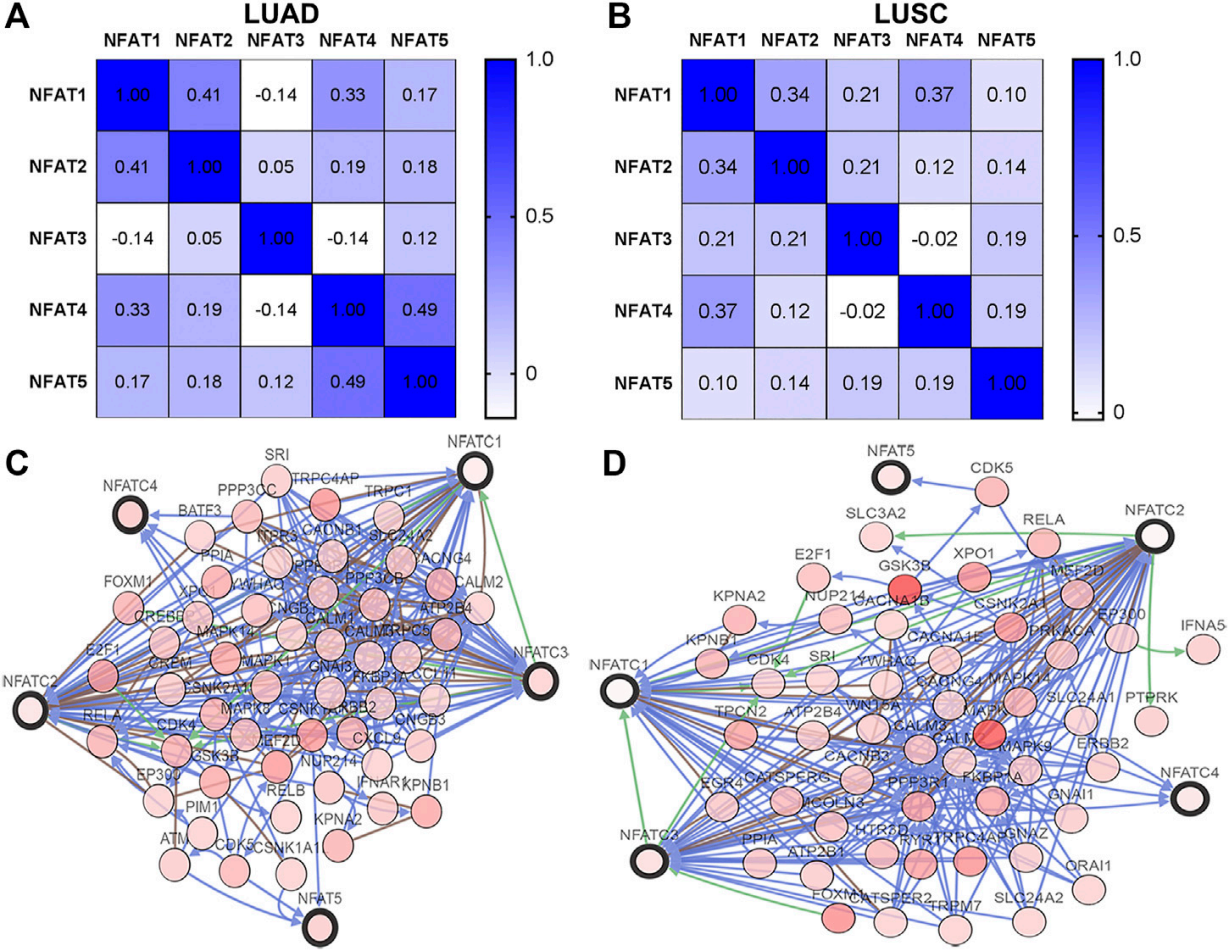
Correlations between NFAT family members and the networks of NFATs and their 50 most frequently altered neighboring genes in LUAD and LUSC analyzed using the cBioPortal database The Pearson correlation coefficients between NFAT family members in LUAD **(A)** and LUSC **(B)** were obtained from the cBioportal database. The networks constructed in the cBioportal showed the interaction relationship between the five NFAT family members (bold black outline) and their 50 most frequently altered neighbor genes (thin black outline) in LUAD **(C)** and LUSC **(D)**. Lung adenocarcinoma, LUAD; lung squamous cell carcinoma, LUSC.

Using the cBioPortal database, we analyzed the Spearman’s correlations between NFAT family members in LUAD and LUSC. In LUAD, there was a positive correlation between NFAT1 and NFAT2 (r = 0.41, *p* = 3.64E-22), NFAT1 and NFAT4 (r = 0.33, *p* = 1.36E-14) NFAT4 and NFAT5 (r = 0.49, *p* = 3.66E-33) (Figure 8A). In addition, the results shown in Figure 8B indicated a positive correlation between NFAT1 and NFAT2 (r = 0.34, *p* = 8.31E-15), NFAT1 and NFAT4 (r = 0.37, *p* = 6.81E-18). The networks for NFAT family members and their 50 most frequently altered neighbor genes in LUAD and LUSC were constructed and displayed in Figures 8C,D, respectively.

### GO Functional Annotation of Nuclear Factor of Activated T Cells and Their 50 Most Frequently Altered Neighboring Genes in Non-small Cell Lung Cancer

**FIGURE 9:**
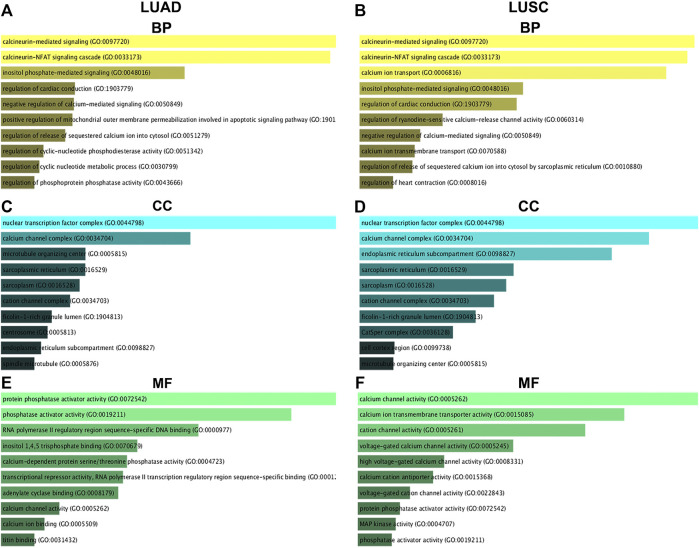
GO functional annotation of NFATs and their 50 most frequently altered neighboring genes in LUAD and LUSC analyzed using the Enrichr database GO functional annotation analysis of NFATs and their 50 most frequently altered neighboring genes was performed by the Enrichr database. The top 10 enriched BP items of NFAT and their 50 most frequently altered neighboring genes in LUAD **(A)** and LUSC **(B)**; the top 10 enriched CC items of NFATs and their 50 most frequently altered neighboring genes in LUAD **(C)** and LUSC **(D)**; the top 10 enriched MF items of NFATs and their 50 most frequently altered neighboring genes in LUAD **(E)** and LUSC **(F)**. Biological process, BP; cellular component, CC; molecular function, MF. Lung adenocarcinoma, LUAD; lung squamous cell carcinoma, LUSC.

The top 10 enriched GO items are listed in Figure 9. Biological process (BP) analysis revealed that NFATs and the genes significantly associated with *NFAT* mRNA expression in LUAD were significantly enriched in calcineurin-mediated signaling, the calcineurin-NFAT signaling cascade, and inositol phosphate-mediated signaling (Figure 9A). For cellular component (CC) analysis, these genes were significantly enriched in the nuclear transcription factor complex, calcium channel complex, and microtubule-organizing center (Figure 9C). The molecular function (MF) analysis of these genes revealed protein phosphatase activator activity, phosphatase activator activity, and RNA polymerase II regulatory region sequence-specific DNA binding (Figure 9E). GO BP analysis demonstrated that NFATs and the genes significantly associated with *NFAT* mRNA expression in LUSC were significantly enriched in calcineurin-mediated signaling, the calcineurin-NFAT signaling cascade, and calcium ion transport (Figure 9B). For CC analysis, these genes were significantly enriched in the nuclear transcription factor complex, calcium channel complex, and endoplasmic reticulum subcompartment (Figure 9D). MF analysis of these genes revealed that they were significantly enriched in calcium channel activity, calcium ion transmembrane transporter activity, and cation channel activity (Figure 9F).

### KEGG Functional Annotation of Nuclear Factor of Activated T Cells and Their 50 Most Frequently Altered Neighboring Genes in Non-small Cell Lung Cancer

**FIGURE 10:**
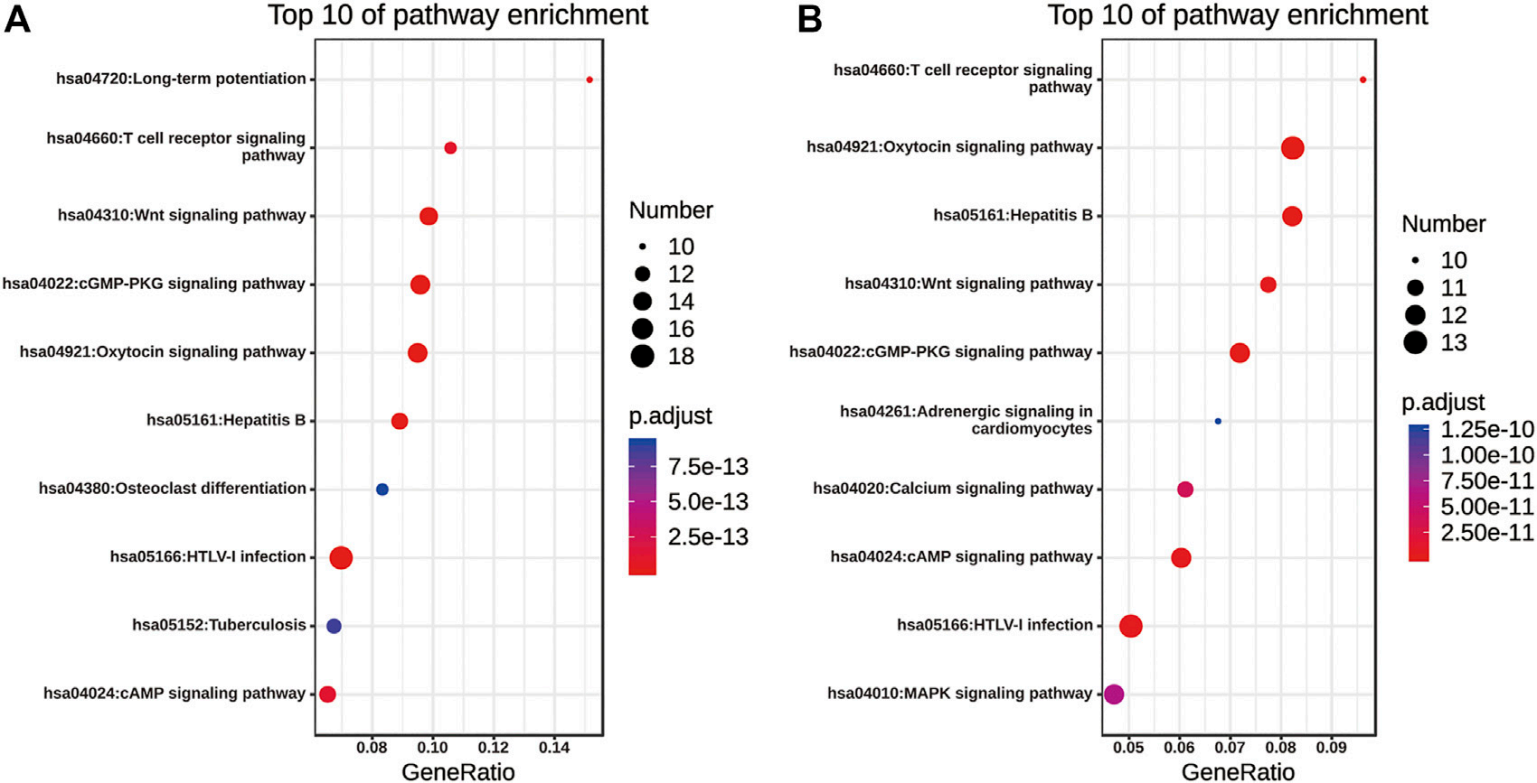
KEGG pathway enrichment of NFATs and their 50 most frequently altered neighboring genes in LUAD and LUSC analyzed using the Enrichr database KEGG pathway enrichment analysis of NFATs and their 50 most frequently altered neighboring genes was performed by the Enrichr database. Bubble plot representing the top 10 enriched KEGG pathways for NFATs and their 50 most frequently altered neighboring genes in LUAD **(A)** and LUSC **(B)**. Y-axis: name of the signaling pathway; X-axis: percentage of the number of genes assigned to a term among the total number of genes annotated in the network; Bubble size: number of genes assigned to a pathway; Color: enriched *p*-value; Red bubble: indicates a greater significance level. Lung adenocarcinoma, LUAD; lung squamous cell carcinoma, LUSC.

The pathway enrichment analyses of NFATs and their 50 most frequently altered neighboring genes in LUAD and LUSC were performed using the Enrichr database. The top 10 of pathway enrichment are listed in Figure 10. As shown in Figure 10A, NFATs and their 50 most frequently altered neighboring genes in LUAD were significantly enriched in human T-lymphotropic viruses, type I (HTLV-I) infection; the cGMP-dependent protein kinase, oxytocin, cAMP, T-cell receptor, and Wnt signaling pathways; hepatitis B; long-term potentiation; osteoclast differentiation; and tuberculosis. Among these pathways, the Wnt signaling pathway was involved in the tumorigenesis and pathogenesis of LUAD. The genes related to the Wnt signaling pathway included glycogen synthase kinase-3 beta (*GSK3B*), E1A binding protein P300 (*EP300*), CREB-binding protein (*CREBBP*), casein kinase 2 alpha 1 (*CSNK2A1*), *CSNK1A1*, *NFAT1*, *NFAT2*, *NFAT3*, *NFAT4*, mitogen-activated protein kinase 8 (*MAPK8*), calcineurin (*PPP3CC*), calcineurin B (*PP3R1*), calcineurin A beta (*PPP3CB*), and casein kinase 1 alpha 1 like (*CSNK1A1L*) were significantly related to NFAT alterations in LUAD. As shown in Figure 10B, the enriched pathways for NFATs and their 50 most frequently altered neighboring genes in LUSC were the oxytocin, cGMP, cAMP, T-cell receptor, calcium, MAPK, and Wnt signaling pathways; hepatitis B; HTLV-I infection; and adrenergic signaling in cardiomyocytes. Wnt signaling pathway-related genes included *MAPK9*, *GSK3B*, *PPP3R1*, *CSNK2A1*, *WNT5A*, *NFAT4*, *NFAT1*, *EP300*, *NFAT2*, *PRKACA*, and *NFAT3* were significantly related to NFAT alterations in LUSC.

## Discussion

Although the dysregulation of one or more NFAT family members has been partially reported in NSCLC, bioinformatics analysis of NFATs in NSCLC has yet to be performed. The present study is the first to evaluate the mRNA expression patterns, potential regulatory networks, and distinct prognostic values of *NFAT* family members in NSCLC through various large databases, including the Oncomine, UCSC Xena Browser and UALCAN databases, Kaplan–Meier Plotter, cBioPortal, and Enrichr. We hope that this study will contribute to systematically discover the role of NFATs in NSCLC, thereby providing candidate targets for the diagnosis and treatment of NSCLC.

*NFAT1* is highly expressed in HCC [31] and breast cancer [32]. Furthermore, it promotes breast cancer cell invasiveness by upregulating ETS proto-oncogene 1 expression [33]. In addition, NFAT1 acts as a novel transcription factor for the oncogene murine double minute 2 (MDM2) that is involved in the regulation of cell proliferation, cell cycle control, and apoptosis [31], suggesting the role of NFAT1 in cancer development, progression, and therapy. However, Ding et al. [12] reported the direct stimulation of NFAT1 by tumor suppressor gene p53 in the HCT116 colon cancer cell line, indicating the protective role of NFAT1 in preventing colon cancer progression. Moreover, colon cancer patients with higher *NFAT1* expression have significantly better OS [12]. Additionally, NFAT1 was also found to act as a tumor suppressor gene to block cell cycle progression by directly regulating cyclin E expression in B lymphocytes [34]. Regarding NSCLC, Chen et al. [25] reported that basal NFAT1 protein expression was upregulated in NSCLC tissues compared with adjacent normal lung tissues, and higher *NFAT1* expression was correlated with the poor prognosis of NSCLC patients. However, in our study, the Oncomine and UALCAN datasets revealed that *NFAT1* mRNA expression was decreased in patients with both LUAD and LUSC in all stages. Furthermore, low mRNA expression of NFAT1 led to a worse OS in patients with LUAD and LUSC, particularly for stage 1, indicating the tumor suppressor role of NFAT1 in NSCLC.

NFAT2 is activated in several malignant tumors including Burkitt lymphoma [35], pancreatic cancer [36], colorectal carcinoma [11], and breast cancer [8]. In addition, Wang et al. [37] reported that *NFAT2* is overexpressed in HCC and promotes the proliferation of HepG2 cell lines, and Xu et al. [7] indicated that *NFAT2* is frequently inactivated in HCC and functions as a tumor suppressor gene to promote the apoptosis of HCC cells by activating the FasL-mediated extrinsic signaling pathway. Moreover, other studies have suggested the tumor suppressor role of NFAT2 in some types of cancer, including human lymphomas [38], chronic lymphocytic leukemia [39], colorectal cancer [40], and squamous skin cancer [41]. Regarding NSCLC, Zhang et al. [42] reported that NFAT2 can function as a tumor suppressor gene to induce the apoptosis of A549 cells. Consistently, Heim et al. [15] reported the significant decrease of *NFAT2* mRNA expression in the solid tumor region of patients with LUAD and LUSC. Furthermore, *NFAT2* mRNA expression in the tumoral area decreased as the tumor stadium progressed [15]. Downregulation of *NFAT2* mRNA expression in the tumoral region correlated with poor prognosis in NSCLC patients [15]. However, Chen et al. [25] found that NFAT2 protein expression was significantly increased in the lung tumor tissues of patients with NSCLC. Furthermore, in NSCLC cell lines, including NCI-H1703, A549, and NCI-H1299, pharmacological inhibition of NFAT2 or NFAT2 knockdown results in growth inhibition [43]. In the present study, we found that *NFAT2* mRNA expression was decreased in patients with LUAD and LUSC in all stages. Furthermore, low *NFAT2* expression was significantly associated with a worse OS in LUAD and LUSC patients. These results raise an interesting question regarding the dual functions of NFAT2 in NSCLC: is oncogenic or tumor-suppressive?

NFAT3 is highly expressed in breast cancer patients, and its knockdown reduces the growth of breast cancer cells [44]. Similarly, NFAT3 is found in various skin cancer cell lines and tumor tissues, and knockdown inhibits tumor cell proliferation, colony formation, and anchorage-independent cell growth in skin cancer cell lines [45]. Consistently, Hessmann et al. [46] indicated that NFAT3 is involved in the development and progression of pancreatic cancer. Interestingly, Gopinath et al. [22] noted that NFAT3 is a prerequisite for doxorubicin-mediated apoptosis, migration, and invasion in SNB19 and U87 glioma cell lines, suggesting that it has a tumor suppressor role in glioma. In this study, we firstly found that *NFAT3* mRNA levels were significantly upregulated in patients with both LUAD and LUSC, particularly for stage 1 and 2. Moreover, LUAD with overexpression of *NFAT3* had a significantly worse OS, indicating the oncogenic role of NFAT3 in LUAD. As little research has focused on NFAT3, the underlying role of NFAT3 in NSCLC needs more investigation.

Knockdown of NFAT4 inhibits the growth of HCT116 cells and tumor xenograft growth in nude mice and is associated with the upregulation of p53 and downregulation of MDM2 [12]. In addition, NFAT4 overexpression is found in gastric cancer tissues, and NFAT4 promotes the proliferation of gastric cancer cells by regulating c-Myc [47]. However, Lee et al. [23] indicated that NFAT4 deficiency in female mice led to the development of aggressive mammary adenocarcinoma, suggesting that NFAT4 might function as a tumor suppressor gene in mammary adenocarcinoma. Regarding NSCLC, Chen et al. [25] reported that basal NFAT4 protein expression was upregulated in NSCLC tissues. However, Pintarelli et al. [48] found that *NFAT4* mRNA expression was downregulated in LUAD. Consistently, the present study demonstrated that the mRNA level of *NFAT4* was significantly decreased in patients with both LUAD and LUSC. Furthermore, patients with the lower mRNA expression level of *NFAT4* had a worse OS, particularly for stage 2, suggesting that NFAT4 potentially represents a novel prognostic marker for OS in LUAD. The results of our studies suggest that NFAT4 has a tumor suppressor role rather than an oncogenic role in NSCLC, but this theory needs further investigation.

Among the five NFAT family members, NFAT5 has been the most studied in LC. Meng et al. [49] reported that miR-194 inhibits the proliferation, migration, and invasion of NSCLC cells (A549 and H1299) through targeting NFAT5. Using in silico software, Rahman et al. [50] identified that NFAT5 may regulate migration and invasion, and serve as a potential therapeutic target in patients with LC. Additionally, Mijatovic et al. [51] noted that UNBS1450-mediated anti-tumor activity was associated with the downregulation of NFAT5 in A549 NSCLC cells. Consistently, NFAT5 protein expression was upregulated in the NSCLC cell lines A549 and H1975, and NFAT5 promoted the proliferation and migration of A549 and H1975 cell lines by regulating the expression of Aquaporin-5 that is involved in the proliferation, metastasis, and invasion of LC [14]. Furthermore, Cho et al. [52] revealed that higher NFAT5 protein expression led to a poor DFS in NSCLC patients who underwent surgical resection. These studies suggest that NFAT5 had an oncogenic role rather than a tumor suppressor role in NSCLC. This is in agreement with our prognostic results that higher mRNA expression of *NFAT5* led to a poorer OS in LUSC. Interestingly, in patients with LUSC, it was found to be downregulated in the UALCAN database. Additionally, the downregulation of *NFAT5* in LUAD in the UALCAN database was supported by the results that higher mRNA of *NFAT5* in LUAD was associated with tumor stage 2 and a better outcome. Taken together, NFAT5 may have complex roles in the pathogenesis and progression of NSCLC. Further studies with larger sample sizes should be carried out to validate the expression of NFAT5 in NSCLC.

To clarify the mechanisms of function of NFAT family members in LUAD and LUSC, we constructed the networks for NFAT family members and their 50 most frequently altered neighbor genes. The results of the functional analysis indicated that these genes are mainly involved in various cancer-related signaling pathways, such as Wnt signaling pathway, thereby affecting the tumorigenesis and progression of NSCLC. The correlation analyses of NFAT family members were also performed in this study. The results suggested that NFAT interactions may play an important role in the pathogenesis and development of NSCLC.

## Conclusion

In LUAD and LUSC, the mRNA expression levels of *NFAT1/2/4/5* were downregulated and *NFAT3* mRNA expression level was upregulated. 16.72% of LUAD and 7.86% of LUSC clinical cases had the mRNA dysregulation of *NFAT*s which was the single most important factor for the alterations in NFATs. *NFAT3* mRNA was the most frequently upregulated in LUAD (∼8%), which suggests its important role in NSCLC. this comprehensive bioinformatics analysis revealed that the NFAT family members display varying degrees of abnormal expressions in LUAD and LUSC, suggesting that these transcription factors may be therapeutic targets for patients with NSCLC. Our data also implied that NFAT1/2/5 could act as promising prognostic biomarkers for LUAD and LUSC and that NFAT3/4 might be new biomarkers for the prognosis of LUAD. These findings would contribute to a better understanding of the distinct roles of NFATs in NSCLC. However, considering currently available limited evidence, the role of NFATs in NSCLC needs further investigation.

## Materials and Methods

### Ethics Statement

The study protocol was approved by Children’s Hospital of Soochow University (Suzhou, China). All the datasets were retrieved from the online databases, so it was confirmed that all written informed consent was obtained.

### Oncomine Analysis

Oncomine Cancer Microarray database (www.oncomine.org) was used to analyze the mRNA expression levels of NFAT family members in different cancers [53]. The mRNA expressions of *NFAT* family in clinical cancer specimens were compared with that in corresponding normal tissues by choosing “Cancer vs. Normal analysis” and “Gene Summary View”, using a Students’ *t*-test to generate a *p* value. The cut-off of *p* value, fold change and gene rank were defined as 0.01, 2 and top 10%, respectively.

### University of California, Santa Cruz Xena Browser Analysis

The LUAD (*n* = 706) and LUSC (*n* = 626) cohorts in TCGA database were obtained from the UCSC Xena browser (http://xenabrowser.net/) which provides a securely analyzed and visualized functional genomic dataset in TCGA datasets. The UCSC Xena browser was utilized to obtain the hierarchical clustering and the corresponding box plots of the mRNA expression levels of *NFAT* family members between Solid Tissue Normal and Primary Tumor (LUAD or LUSC). The UCSC Xena browser analyzed the data from the TCGA database which was different from the Oncomine database.

### UALCAN Analysis

UALCAN (http://ualcan.path.uab.edu/), a web resource, provides a comprehensive cancer transcriptome data from 31 cancer types of TCGA database [54]. The expression analysis was based on clinical characteristics of cancer patients, including individual cancer stages, tumor grade, gender, age, and other clinicopathological features. *NFAT* transcription in subgroups of LUAD and LUSC patients based on individual pathological cancer stage and smoking habits was analyzed.

### cBioPortal Analysis

The LUAD (TCGA, Provisional) dataset including data of 585 samples and LUSC (TCGA, Provisional) dataset including data of 511 samples, were selected for the analyses of alteration frequency and network of NFAT using the cBioPortal (www.cbioportal.org) that provides visualization, analysis, and download of large-scale cancer genomics datasets for TCGA [55, 56]. The genomic profiles included mutations (Missense Mutation and Truncating Mutation), copy-number variance from GISTIC (Amplification and Deep Deletion), mRNA expression (mRNA High and mRNA Low) z-scores (RNA Seq V2 RSEM) with a z score threshold of ±2.0, and protein expression z-scores (RPPA), which indicates the distinct types of alternations in NFAT isoforms. According to the cBioPortal’s online instruction, the network was constructed, and OS and DFS/PFS were calculated. Additionally, this database was used to evaluate the correlations between NFAT family members. The Spearman’s correlation coefficient with >0.3 or < −0.3 indicated a good correlation.

### Kaplan-Meier Plotter Analysis

The prognosis associated with the NFAT family members was analyzed using the Kaplan-Meier plotter (http://kmplot.com/analysis/) which contained gene expression data and survival information of 3452 clinical lung cancer patients from the databases include Gene Expression Omnibus database, European Genome-phenome Archive, and TCGA [57]. The patients with LUAD or LUSC were divided into two groups, high expression group and low expression group, by the median expression values of *NFAT* family members. The prognosis was evaluated by a Kaplan-Meier survival plot, with the hazard ratio with 95% confidence intervals and logrank *p* value. Only the JetSet best probe set of NFATs for mRNA expression or user selected probe set of NFATs for clinico-pathological features were chosen. Logrank *p* < 0.05 was considered as statistically significant.

### GO Annotation and KEGG Pathway Enrichment Analysis

The Enrichr database (http://amp.pharm.mssm.edu/Enrichr/) was utilized to perform GO functional annotation and KEGG pathway enrichment analysis for NFAT and genes significantly associated with *NFAT* mRNA expression [58]. The GO analysis included three categories, namely, BP, CC, and MF. Adj. *p* < 0.05 was considered to be statistically significant.

## Data Availability

Publicly available datasets were analyzed in this study, these can be found in the Oncomine Cancer Microarray database (www.oncomine.org) and TCGA database were obtained from the UCSC Xena browser (http://xenabrowser.net/), UALCAN (http://ualcan.path.uab.edu/) and the cBioPortal (www.cbioportal.org).
